# The Gap between Individual Perception and Compliance: A Qualitative Follow-Up Study of the Surgical Safety Checklist Application

**DOI:** 10.1371/journal.pone.0149212

**Published:** 2016-02-29

**Authors:** Gerald Sendlhofer, David Benjamin Lumenta, Karina Leitgeb, Brigitte Kober, Lydia Jantscher, Monika Schanbacher, Andrea Berghold, Gudrun Pregartner, Gernot Brunner, Christa Tax, Lars Peter Kamolz

**Affiliations:** 1 Executive Department for Quality and Risk Management, University Hospital Graz, Graz, Austria; 2 Division of Plastic, Aesthetic and Reconstructive Surgery, Department of Surgery, Medical University of Graz, Graz, Austria; 3 Research Unit for Safety in Health, Medical University of Graz, Graz, Austria; 4 Division of Oral and Cranio-Maxillofacial Surgery, Department of Dentistry and Maxillofacial Surgery, Medical University of Graz, Graz, Austria; 5 Institute for Medical Informatics, Statistics and Documentation, Medical University of Graz, Graz, Austria; 6 University Hospital Graz, Graz, Austria; Stanford University School of Medicine, UNITED STATES

## Abstract

**Background:**

“*The Surgical Safety Checklist* (*SSC) is important*, *but we don’t use it adequately*” is a well-suited statement that reflects the SSC's application in hospitals. Our aim was to follow up on our initial study on compliance (2014) by analysing differences between individual perception and compliance with the SSC.

**Methods:**

We conducted a follow-up online survey to assess healthcare professionals’ individual perception of, as well as satisfaction and compliance with the SSC three years following its thorough implementation.

**Results:**

171 (19.5%) of 875 operating team members completed the online survey. 99.4% confirmed using the SSC. Self-estimated subjective knowledge about the intention of the checklist was high, whereas objective knowledge was moderate, but improved as compared to 2014. According to an independent audit the SSC was used in 93.1% of all operations and among the SSCs used the completion rate was 57.2%. The use of the SSC was rated as rather easy [median (IQR): 7 (6–7)], familiar [7 (6–7)], generally important [7 (7–7)], and good for patients [7 (6–7)] as well as for employees [7 (7–7)]. Only comfort of use was rated lower [6 (5–7)].

**Conclusion:**

There is a gap between individual perception and actual application of the SSC. Despite healthcare professionals confirming the importance of the SSC, compliance was moderate. The introduction of SSCs in the health care sector remains a constant challenge and requires continuous re-evaluation as well as a sensible integration into existing workflows in hospitals.

## Introduction

Within the University Hospital Graz, the Surgical Safety Checklist (SSC) has been in use since 2011, and was thoroughly implemented across the entire hospital in 2012. Recently published data on our SSC-use demonstrated that further improvements were needed to achieve better compliance and acceptance of the SSC [1]. It became obvious that the general use of the SSC was good, however, the rate of completed SSCs continuously decreased over time.

On the one hand, similar results were observed in an observational study in Colorado, where 90% of hospitals used the SSC, but reported inconsistent and incomplete use [2]. On the other hand, inadequate use did not influence a 30-day measure of mortality [3–6]. Other studies showed that the correct use of the SSC reduced complications [5, 7, 8]. Haugen and colleagues also reported a dosage-like effect on the completion rate of SSCs, where a significant reduction of major complications occurred when teams completed all checklist items [9].

Some employees still feel that the SSC is just a tick-off list and monitoring tool [10]. We have to understand that the SSC is a new tool that has to be integrated into a habitual process that has not much changed in the past. Therefore, it is imperative that the SSC has to be adapted to local circumstances. The overall intention of the SSC is to create an environment empowering all team members to speak up and discuss every item, although this component is sorely neglected so far [2, 10–12]. One reason might be the hierarchical barriers present in an OR [13]. Therefore, apart from adapting the SSC to local circumstances with all stakeholders, an important factor in reaching SSC acceptance and compliance is promotion, training and specific feedback mechanisms to improve individual knowledge, compliance and thereby the intention for SSC-usage [1, 13, 14].

So far, SSC-implementation has also had little impact on changing the safety climate [15]. Up to now, simply making the SSC mandatory has not led to the expected outcome of reducing risks for patients [2], and non-adherence to internal guidelines seemed to be a commonly accepted practice. A Swiss study showed that both surgeons and anaesthetists perceived the SSC as a valuable instrument in general and that opinions towards the SSC were overall positive. However, the study also revealed that one of the main intentions of the SSC—achieving better teamwork—was not being facilitated by the tool [16]. Rydenfält therefore postulated that personnel’s conception and perceived importance of SSC items predominantly influences SSC application [11].

Another study indicated that physicians would want the SSC to be used if they were to undergo surgery themselves; however, the same professional group was still resistant to using it as proposed [10]. Taking the patients’ view, the majority also agreed that they would like the SSC to be used and that it would make them feel safer. The fact that the tool had just been introduced in the healthcare sector was met with surprise [17].

In the University Hospital of Graz, efforts were made to facilitate the SSC among all healthcare professionals. To increase SSC-compliance we offered a bundle of interventions. First, corresponding operating teams received an introduction through a YouTube video from the NHS entitled “How not to do the surgical safety checklist”. Secondly, in bespoke role plays, employees were trained on how to ask and answer questions. Third, we regularly performed systematic audits and gave feedback on SSC-compliance in focus group discussions. Finally, all employees working in an OR were asked through a survey to give us their feedback on professionals’ knowledge of, satisfaction with, as well as individual perception of SSC-usage [1]. The baseline survey in 2014 revealed promising results; however, internal audits showed contrary findings. Subsequently, we offered further individual training sessions for each OR-team; nevertheless, these sessions were not sought after by senior managers as expected. Additionally, within the baseline survey, annotations were given and healthcare professionals suggested an in-house-training video to further support SSC-training. Therefore, a training video supporting adult-based learning and demonstrating the correct use of the SSC was produced as a supporting tool to gain progress in increasing the intention of SSC-usage [10, 18].

This follow-up study primarily aimed to examine if further promotion of the SSC positively influenced healthcare professionals’ individual perception of the SSC. The secondary aim was to re-assess healthcare professionals’ knowledge of, as well as satisfaction and compliance with the SSC three years after its thorough implementation.

## Materials and Methods

The study (online survey) was approved by the Medical University Graz Ethics Committee (vote-number: 26–137 ex 13/14).

### Training video

An in-house training video was produced [18]. The link to the video was sent out via email to all senior managers in January 2015. Senior managers were asked to show the video to their colleagues in one of their routine meetings. Additionally, the link to the video was sent to all healthcare professionals working in an OR. The use of the educational video was tracked based on how many times it was accessed via YouTube channel.

### Assessing compliance

According to the first study by Sendlhofer et al., unannounced audits were introduced to assess SSC-compliance rates. Altogether, four audits took place (February and November 2013, June 2014, May 2015) [1]. After finalizing the follow-up online survey in May 2015, two additional days were determined for the fourth audit and announced via email. The Department of Quality and Risk Management collected the SSCs. The number of operations performed versus the number of collected SSCs was compared with scheduled and definitively performed operations. Corresponding data were collected from the hospital’s electronic documentation system [1]. We also performed quantitative analyses to review whether checklist items were ticked or not. If one checklist item was not ticked, the SSC was rated as incomplete.

### Online survey

As done in 2014, from April 15^th^ to May 14^th^ 2015 we used a validated survey for online assessment of frequency of use, satisfaction with the implementation as well as subjective and objective knowledge (S1 and S2 Tables). Additionally within this follow-up study, we examined individual perception of the SSC's usefulness by asking 6 questions, which were polled in the initial survey in 2014 (data previously not published). For SSC-usefulness, a 7-point-Likert-scale ranging from “do not agree at all” corresponding to “1” to “completely agree” corresponding to “7” was used.

All employees working in an OR were given the opportunity to provide feedback. The online questionnaire was sent to 875 employees working in one of the 44 operating theatres, a sample that corresponds to 20% of the total workforce of the university hospital and included all professional groups. The procedure of the survey was in analogy to the initial online survey in 2014 [1]. As proposed by Burns et al., weekly reminders were automatically sent by the system to non-responders [19].

### Statistical analysis

Survey data were analysed using descriptive statistics for the total cohort and for each of the two professional groups (consultants and nursing staff). Categorical variables are presented as absolute and relative frequencies; for metric variables, median and interquartile ranges (IQR; range from first to third quartile) are given, since none of these variables was normally distributed. Differences between the professional groups were assessed by Mann-Whitney U or Chi-Squared test according to the variable level. Differences between the surgical and anaesthetic staff within the two professional groups were also tested. Since the study was not hypothesis-driven, all analyses are of a purely exploratory nature. All analyses were conducted using SPSS version 22.

## Results

### Checklist use

**Fig 1:**
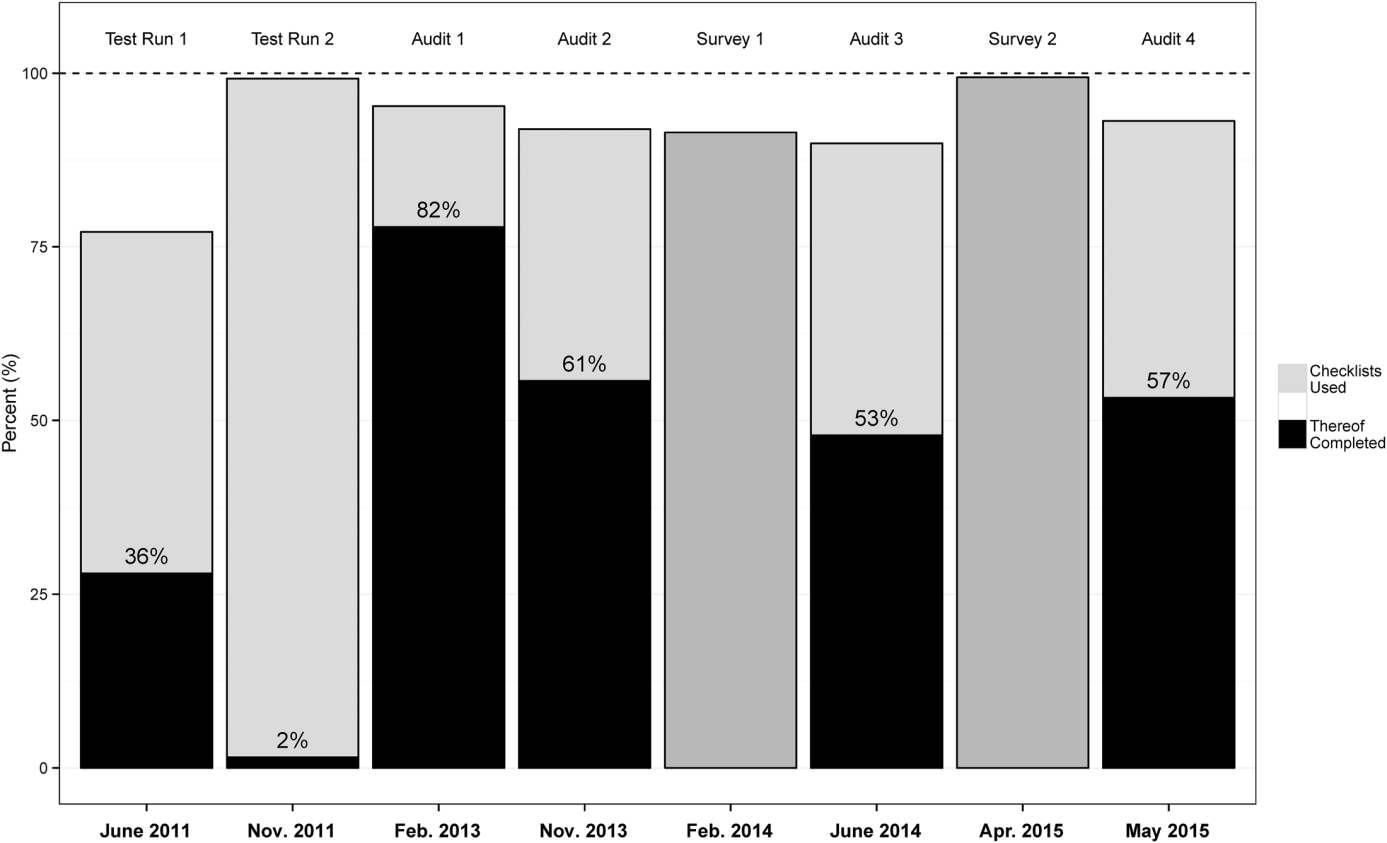
Utilization and completion rates of the SSC. Data up to audit 3 are depicted from Sendlhofer et al. [1].

The fourth unannounced audit showed that SSCs were used in 93.1% (173/291) of operations. Among the SSCs used, 42.8% (116/271) had been partially completed. In the two years between the second and the current audit, completion rates remained similar with 60.6% in the second, 53.2% in the third and 57.2% in the current audit (Fig 1) [1].

### General survey results

In 2015, 875 employees were asked to participate in the follow-up online survey and the overall response rate was 19.5% (Table 1). Compared to the initial survey in 2014, relatively more consultants responded to the survey. The sample consisted of 57.9% (2014: 66.9%) women. The median age was 40 (2014: 40.5). 60.8% (2014: 58.8%) of responders had been working in an operating theatre for at least 10 years and 53.2% (2014: 56.9%) revealed having spent at least 32 hours per week there.

**Table 1:** Online survey—sample characteristics.

Survey (year)	2014*	2015	2014*	2015
	Consultants	Consultants	Nursing staff	Nursing staff
N (%)	60 (37.5)	76 (44.4%)	100 (62.5)	95 (55.6%)
Female (%)	28 (46.7)	24 (31.6)	79 (79.0)	75 (78.9)
Median age in years (range)	46.5 (28, 59)	47 (28, 60)	36 (21, 60)	33 (20, 62)
Professional experience (%)				
0–< 5 years	6 (10.0)	8 (10.5)	21 (21.0)	30 (31.5)
5–< 20 years	38 (63.3)	46 (60.5)	52 (52.0)	42 (44.2)
More than 20 years	16 (26.7)	22 (28.9)	27 (27.0)	23 (24.2)
Hours spent in the OR in an average week				
0–< 16	12 (20.0)	27 (35.5)	21 (21.0)	11 (11.6)
16–< 32	11 (18.3)	22 (28.9)	25 (25.0)	20 (21.1)
32–and more than 40	37 (61.7)	27 (35.5)	54 (54.0)	64 (67.4)

* data are depicted from Sendlhofer et al. [1]

### Frequency of checklist utilization

99.4% (2014: 91.3%) stated that they used the SSC and 88.3% (2014: 80.6%) thereof specified having used the SSC in 91–100% of all operations (Table 2). Compared to the initial survey, in three out of four professional groups relatively more participants revealed using the SSC in 91–100% (S1 Fig). Overall, the estimation of consultants versus nursing staffs regarding how often they were using the SSC did not differ significantly (p = 0.108). Further analysis in the respective subgroups did not demonstrate any significant differences between surgeons and anaesthesiologists (p = 0.212) nor between nurse anaesthetists and operating theatre nurses (p = 0.451).

**Table 2:** Results of the online survey.

	2014*	2015	2014*	2015
	Consultants	Consultants	Nursing staff	Nursing staff
	N = 60 (37.5%)	N = 76 (44.4)	N = 100 (62.5%)	95 (55.6)
Use of SSC (%)				
Yes	59 (98.3)	75 (98.7)	87 (87.0)	95 (100.0)
Frequency of SSC use				
0–70% of operations	1 (1.7)	3 (4.0)	14 (14.0)	6 (6.4)
71–90% of operations	6 (10.0)	2 (2.7)	10 (10.0)	8 (8.4)
91–100% of operations	53 (88.3)	70 (93.3)	76 (76.0)	81 (85.3)
Satisfaction with SSC				
Very satisfied and satisfied	45 (75.0)	53 (70.7)	54 (54.0)	52 (54.7)
Somewhat satisfied	9 (15.0)	13 (17.3)	30 (30.0)	31 (32.6)
Rather and very unsatisfied	6 (10.0)	9 (12.0)	16 (16.0)	12 (12.6)
Subjective knowledge				
Very good and rather good	50 (83.3)	61 (80.3)	82 (82.0)	91 (95.8)
Okay	9 (15.0)	12 (15.8)	10 (10.0)	3 (3.2)
Rather bad and very bad	1 (1.7)	3 (3.9)	8 (8.0)	1 (1.1)
Median number of correctly answered questions (range)	8 (4, 10)	8 (4, 10)	8 (3, 9)	8 (4, 10)

* data are depicted from Sendlhofer et al. [1]

### Satisfaction with checklist implementation

Satisfaction with the implementation of the SSC decreased among the professional groups of consultants and remained almost the same for nursing staff (Table 2). However, it can be seen that within nursing staff, operating theatre nurses are now less frequently satisfied or very satisfied, whereas satisfaction among nurse anaesthetists actually increased (S2 Fig). Overall, the satisfaction of consultants and nursing staff with the SSC implementation process did not differ significantly (p = 0.072). No differences were found between surgeons and anaesthesiologists (p = 0.202) or between anaesthetic and operating theatre nurses (p = 0.352).

### Knowledge concerning the SSC

Self-reported knowledge about the SSC and its usage was high among all professional groups. 88.9% (2014: 82.5%) stated that their knowledge was either very good or rather good (Table 2). Whereas surgeons were less confident about their knowledge than in 2014, all other professional groups indicated more frequently having rather good or good knowledge (S3 Fig). Consultants’ and nursing staffs’ estimation of their subjective knowledge did not differ significantly (p = 0.487). There were also no observed differences between surgeons and anaesthesiologists (p = 0.739) or between the two subgroups of nursing staff (p = 0.231).

With regard to objective knowledge, measured as the total number of correctly answered true/false questions, a median of 8 out of 10 questions (IQR: 7–9) were answered correctly and these numbers were identical among consultants (median: 8; IQR: 7–9) and similar for nursing staff (median: 8; IQR: 7–8), but with significant differences between consultants and nursing staff (p = 0.041) (S4 Fig). As in the initial study, no significant correlation between subjective and objective knowledge was found (data not shown).

### Individual perception of the SSC's usefulness

The estimation of individual perception of the SSC's usefulness, measured on a 7-point Likert scale, showed that the use of the SSC was rated as rather easy [median (IQR): 7 (6–7) in both 2014 and 2015], familiar [7 (6–7) in both years], generally important [7 (7–7) in both years], and good for patients [7 (6–7) in both years] as well as for employees [7 (7–7) in both years]. Only comfort of use was rated lower [6 (5–7) in both years]. In 2014, there were no significant differences between the consultants and nursing staff, whereas in 2015 nursing staff rated the use of the SSC as more important (p = 0.007) and better for patients (p = 0.020). Differences between surgeons and anesthesiologists as well as OR nurses and nurse anesthetists are shown in Table 3.

**Table 3:** Median (IQR) individual perception of SSC-usefulness on the 7-point-Likert-scale.

	2014	2014		2015	2015	
The use of the SSC is	Surgeon	Anesthesiologist	**p**	Surgeon	Anesthesiologist	**p**
- easy (= 7) or difficult (= 1)	7 (6–7)	7 (6–7)	0.670	7 (6–7)	7 (6–7)	0.511
- comfortable (= 7) or uncomfortable (= 1)	6 (5–6)	7 (6–7)	0.008	6 (5–7)	6 (5–7)	0.713
- familiar (= 7) or unfamiliar (= 1)	6 (5–7)	7 (6–7)	0.097	7 (6–7)	7 (6–7)	0.612
- important (= 7) or not (= 1)	7 (6–7)	7 (7–7)	0.046	7 (6–7)	7 (7–7)	0.486
- good (= 7) for employees or not (= 1)	7 (6–7)	7 (7–7)	0.028	7 (6–7)	7 (6–7)	0.849
- good (= 7) for patients or not (= 1)	7 (6–7)	7 (7–7)	0.046	7 (6–7)	7 (6–7)	0.893
	OR Nurse	Nurse anesthetists		OR Nurse	Nurse anesthetists	
- easy (= 7) or difficult (= 1)	7 (6–7)	6 (5–7)	*0*.*109*	7 (6–7)	6.5 (6–7)	*0*.*133*
- comfortable (= 7) or uncomfortable (= 1)	6 (5–7)	6 (5–6.5)	*0*.*165*	6 (5–7)	6 (5–6)	*0*.*051*
- familiar (= 7) or unfamiliar (= 1)	7 (6–7)	6 (5–7)	*0*.*019*	7 (6–7)	6 (5–7)	*0*.*001*
- important (= 7) or not (= 1)	7 (6–7)	7 (7–7)	*0*.*489*	7 (7–7)	7 (7–7)	*0*.*218*
- good (= 7) for employees or not (= 1)	7 (6–7)	7 (5.5–7)	*0*.*616*	7 (7–7)	7 (6–7)	*0*.*002*
- good (= 7) for patients or not (= 1)	7 (7–7)	7 (7–7)	*0*.*931*	7 (7–7)	7 (7–7)	*0*.*599*

## Discussion

In 2015, results of the fourth audit and the follow-up online survey showed that there is a gap between individual perception and SSC-compliance between healthcare professionals. Whereas healthcare professionals believed that they used the SSC in 99.4% of all operations, the fourth independent audit showed that the SSC was used in 93.1% and completion rates were even lower (57.2%). In contrast to observed compliance rates, our data further indicated that individual perception of the SSC's usefulness was quite high, and revealed differences among professional healthcare groups. Overall, these findings demonstrate a distinction between perceived importance and actual use as well as compliance with the checklist items.

A number of studies have shown that the SSC has been able to reduce complication and mortality rates [5, 6, 8, 9, 13, 20], and reports also recommended full compliance with the SSC as a result of its dosage-dependant response on SSC-completion and complication rates [6, 9, 21]. Nevertheless, the use of the SSC is still not a “*fast selling item*” [1, 6]. But what are the reasons for not using the SSC as would be expected and why is there still a need to explain the correct use of a checklist per se?

Prior to the implementation of the SSC in our university hospital, an interdisciplinary team with all healthcare professionals adapted the WHO-SSC in order to best incorporate it into existing operative workflows and to best overcome obstacles and associated fears. Additionally our SSC also offers the opportunity to tick off certain checklist items with either “yes”, “no” or “not relevant” as for instance for checklist items “site marked”, “critical or unexpected steps” or “equipment problems”. This option was implemented for cases where certain checklist items might not be relevant for a particular procedure.

In general SSC-usage was very good, however, completion rates were too low. One reason for poor SSC completion rates might be the fact that the implementation of the SSC was a top-down-driven process. Healthcare professionals still perceive the SSC as a monitoring tool and deviate from their good intentions. All professional groups also admit that the use of the SSC is less comfortable. We have to understand that the SSC is a new tool that has to be integrated into a habitual process. The SSC therefore demands a behavioural change from all team members in an OR and this might further explain why a certain amount of time is need before the envisaged benefit becomes evident.

Within our setting, independent audits of SSC-use started in 2013 and since then it seemed that we have reached a steady state. SSC completion rates varied between 53.2% and 60.6% of used SSCs and are comparable to recently published data from five NHS hospitals [6]. Considering that a large proportion of SSCs were incomplete, perceived importance of checklist items might play a major role in SSC compliance [11]. Therefore, we consistently involve healthcare professionals of each surgical department after each audit to ask whether further changes to the SSC are necessary.

Concerning compliance to checklist items one might argue that specific checklist items might not be relevant for a particular procedure and thus, if they are omitted from the SSC it may not mean that the team is not performing the SSC or not adhering to its intent and content. We have to admit that in our case the SSC offers the opportunity to answer certain checklist items with either “yes”, “no” or “not relevant”. Therefore, we are convinced that the audit of SSCs in terms of completion rates is an appropriate tool to prove compliance to the SSC. Therefore, we are surprised, that the gap between high individual perception and compliance of the SSC still remains *constant at approximately 60%*. Performing audits of SSC checklist items completion rates is an adequate tool to prove compliance. Nevertheless, in order to prove team performance with respect to SSC-usage, audits in an OR are needed to further investigate the possible disconnection between physical completion of the SSC and actual compliance to checklist items.

The risk assessment approach of the aviation industry has provided healthcare with some best practice examples on systematically dealing with medical errors [22]. In aviation there is no doubt about the usefulness of checklists and checks are performed routinely, however without ticking any checkbox items. All professional groups perceive that the SSC is good for patients, is very important and in particular for employees. Though the frequency of checklist use increased and reached almost 100%, none of the professional groups felt fully comfortable when using the SSC. This can most likely be explained by existing hierarchical barriers [23]. At the same time, the valuable progress in SSC-use might be a result of the constant promotion of the SSC, however, SSC-completion in daily routine may still be perceived as inhibitory to the routine workflow.

In the meantime, the SSC is being used worldwide and is broadly accepted [16], nevertheless its application is still questioned with respect to benefits as in non-technical skills. Human factor-based training and structured team training were proposed to support better SSC-compliance [20, 24], however, we observed individual opposition to one-on-one training sessions. Healthcare professionals feel uncomfortable when being supervised by a third party. Nonetheless, customized training proved to be effective as compared to standardized training by simply using videos or posters from third parties [25]. This is why we introduced an in-house training video supporting adult-based learning where healthcare professionals from one department were shown how to best run the SSC [18]. We postulated that through customized training, positive trends towards SSC-individual perception, knowledge and compliance could be reached. The video was sent out to all healthcare professionals working in an OR. We knew that not all healthcare professionals open their emails routinely, and some have never even activated their email account, which is why we also encouraged all senior managers to show the video in their daily routine meetings. Tracked by YouTube, the video was accessed more than 500 times. We believe that it was most often healthcare professionals from the university hospital who accessed the video on YouTube channel, as we did not promote the video to a broader community in Austria. We also know that the video was presented very often during routine meetings; in these cases, one hit on YouTube means that more healthcare professionals had seen the video. In general we observed increased subjective knowledge on checklist use as well as increased objective knowledge. We assume that the in-house-video might have had a certain impact.

To summarize, individual perception and usefulness of the SSC was found to be high and increased in one year, however, when using the SSC as expected, healthcare professionals felt less comfortable. Barriers with respect to low compliance are diverse and can most commonly be triggered by engaged leadership as well as by a checklist that fits into routine procedures. Personnel’s conception of the SSC influences its use [11], even though we observed highly perceived usefulness of the SSC, increased subjective and objective knowledge but less compliance. Therefore we have to raise some questions in the future: i) what were the “blockers” to the introduction and utilisation of the SSC, ii) is there a need for an SSC with checkbox items to tick off or is the approach taken by the aviation industry sufficient, iii) are there still too many checkbox items, iv) did the SSC prevent any harm in the past, and v) what are success factors in SSC-usage?

The study has several limitations. The poor response rate did not improve despite sending out three reminders to non-responders. As already mentioned in the initial survey, employees reported certain concerns regarding anonymity, even though the survey process was outlined in detail. In the future, hard copy rather than online surveys might be helpful in increasing the response rate and reducing scepticism with regard to anonymity. Another reason for the poor response rate could have been the peculiar fact that 25–50% of all employees within our hospital had not yet activated their email account, which was revealed after the initial survey [1]. Therefore, we encouraged senior managers to also informally remind their colleagues to participate in the survey. Due to study design we are not able to prove whether employees who took part in the initial survey also took part in the second survey. The fact that only a certain number of employees had access to their email may have also influenced educational intervention with the in-house video. We were also only able to trace the access to the video via the official YouTube website. More than 500 clicks to the video might also include clicks by anyone else outside the university hospital who found the video on YouTube. Furthermore, within the online survey we did not ask whether employees had accessed the video, therefore we are not able to evaluate if the video-intervention had any effect on the results. Finally we assessed compliance in terms of SSC use and ticking off checklist items, but we did not investigate the quality of the SSC use. Quality measurements within each OR are part of an ongoing process, however data are not available as of yet for all ORs.

## Conclusion

In conclusion, we detected that individual perception of the usefulness of the SSC stands in contrast to its actual application and compliance. Promoting its use and usefulness seems to be an ongoing challenge towards the goal of gaining acceptance amongst healthcare professionals and raising compliance in order to create a safe environment for patients and employees.

## Supporting Information

S1 Fig Comparing frequency of checklist use per specialty group of consultants and nursing staff in 2014 and 2015, numbers above bars represent absolute frequencies. (TIFF)

S2 Fig Comparing satisfaction with checklist implementation per specialty group of consultants and nursing staff in 2014 and 2015, numbers above bars represent absolute frequencies. (TIFF)

S3 Fig Comparing subjective knowledge about checklist use per specialty group of consultants and nursing staff in 2014 and 2015, numbers above bars represent absolute frequencies. (TIFF)

S4 Fig Comparing objective knowledge about checklist use per specialty group of consultants and nursing staff in 2014 and 2015. (TIF)

S1 Table Questionnaire items: general use of the SSC, frequency of SSC use, satisfaction with the implementation, subjective and objective knowledge (correct answer in brackets) as well as individual perception of the SSC’s usefulness as used in the Swiss survey. Question 2 of objective knowledge was changed to adhere to local procedures.
(DOCX)

S2 Table Questionnaire items in the original language as used in the Swiss survey: Wissen und Einstellung zur OP-Checkliste. (DOCX)
