# Supplementary figures and images for: The Gap between Individual Perception and Compliance: A Qualitative Follow-Up Study of the Surgical Safety Checklist Application
Source: PLoS One. 2016 Feb 29;11(2):e0149212. doi: 10.1371/journal.pone.0149212 (PMC4771169; doi:10.1371/journal.pone.0149212)

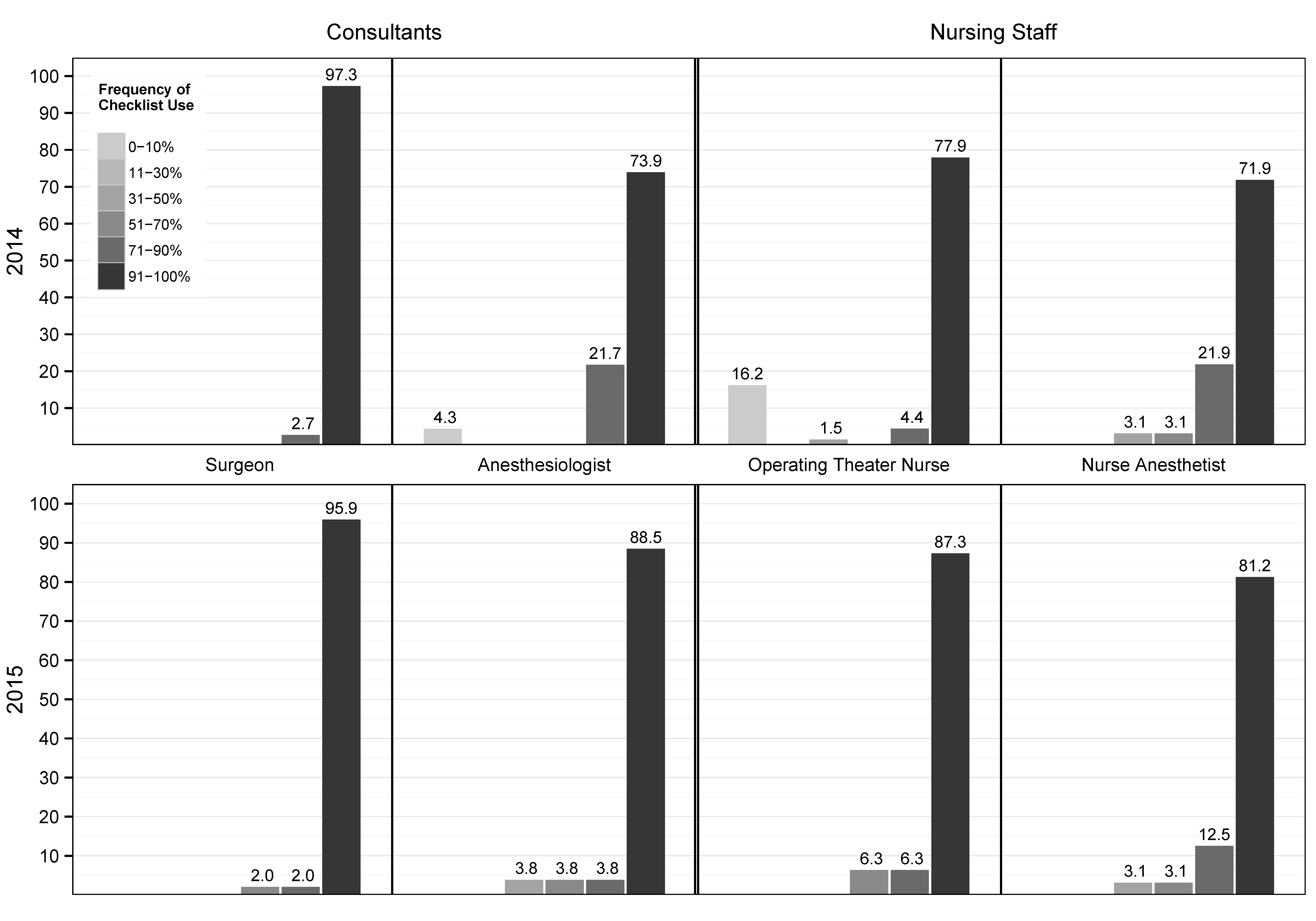

Supplement: S1 Fig — (TIFF) [file pone.0149212.s001.tiff]

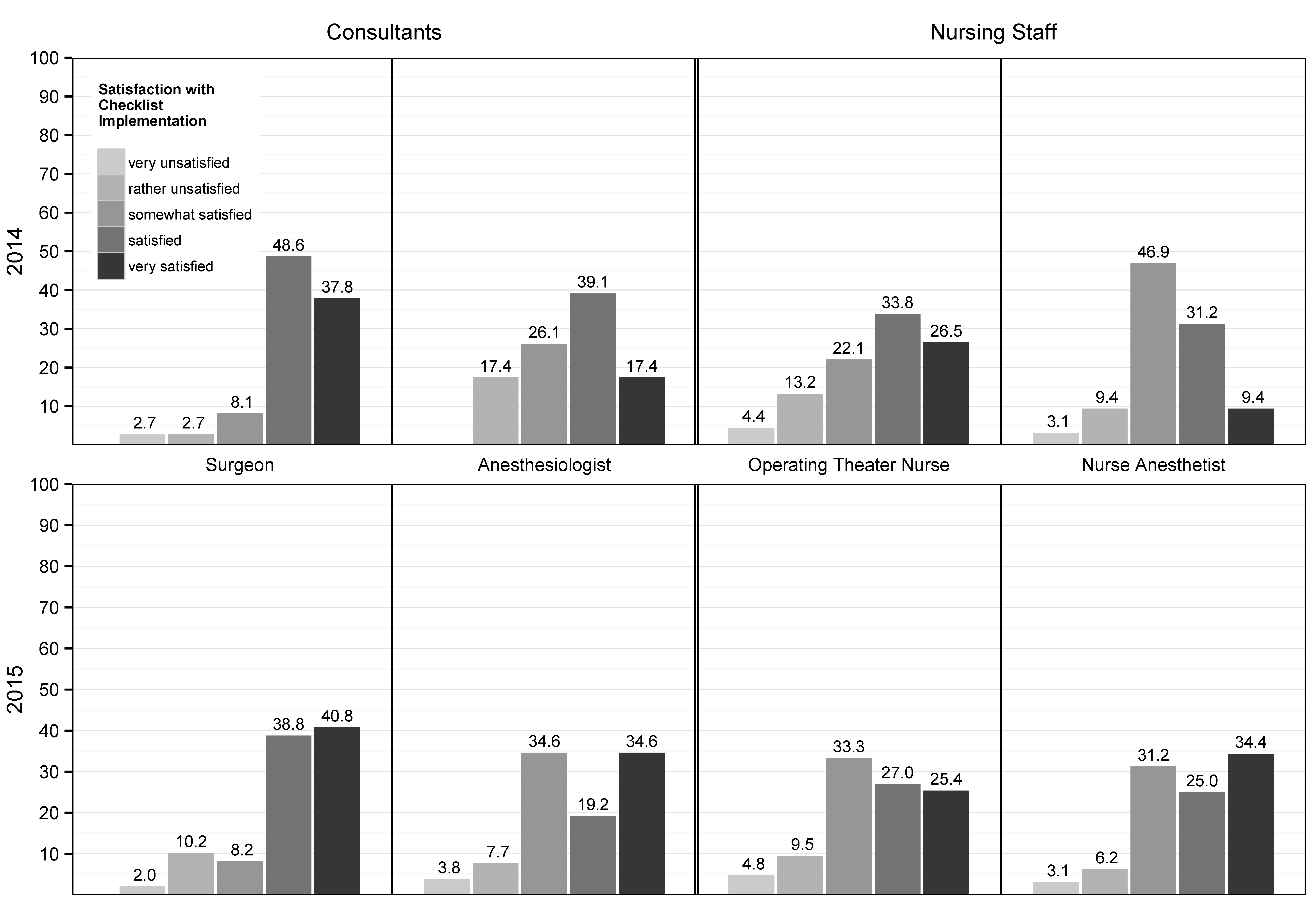

Supplement: S2 Fig — (TIFF) [file pone.0149212.s002.tiff]

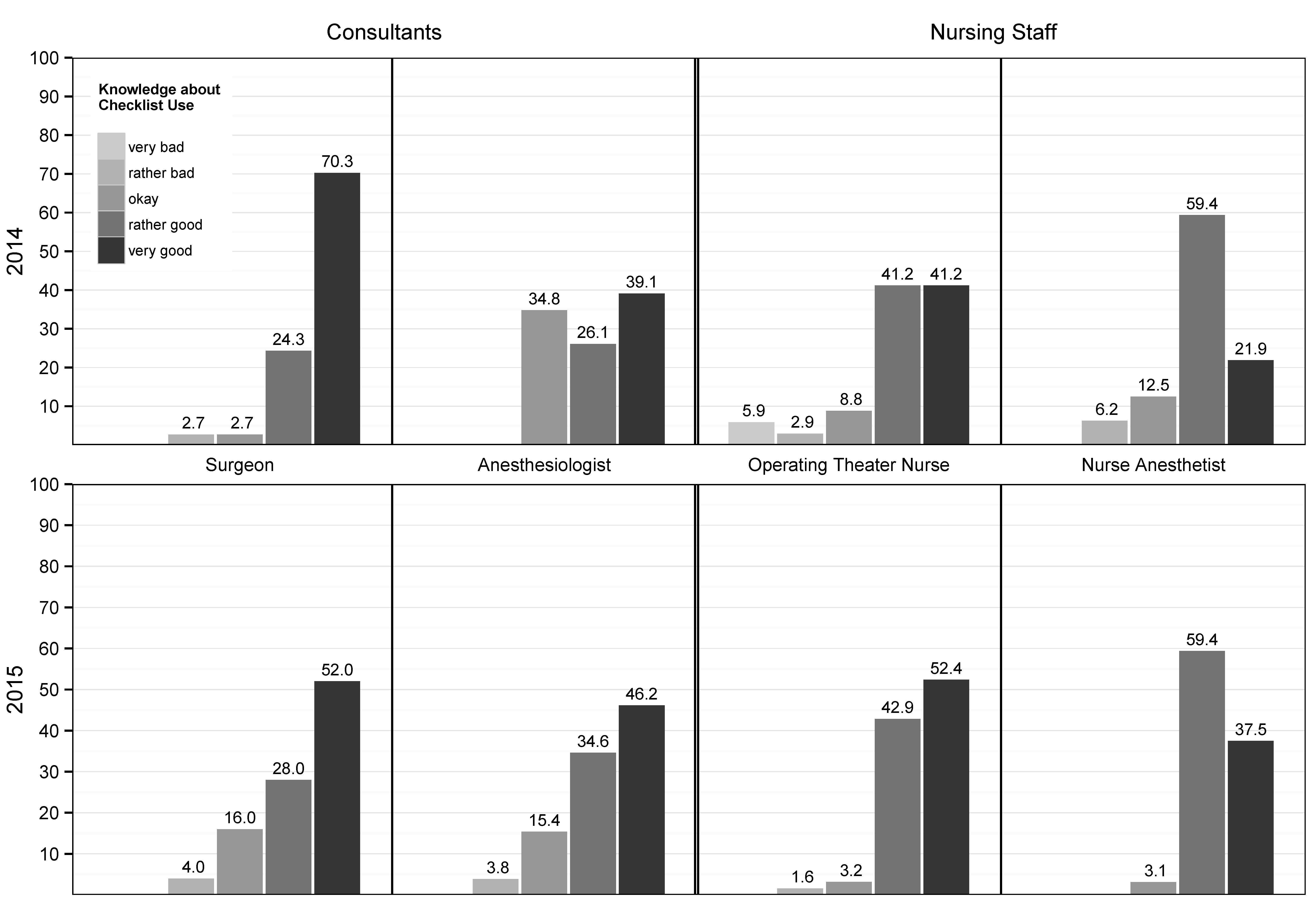

Supplement: S3 Fig — (TIFF) [file pone.0149212.s003.tiff]

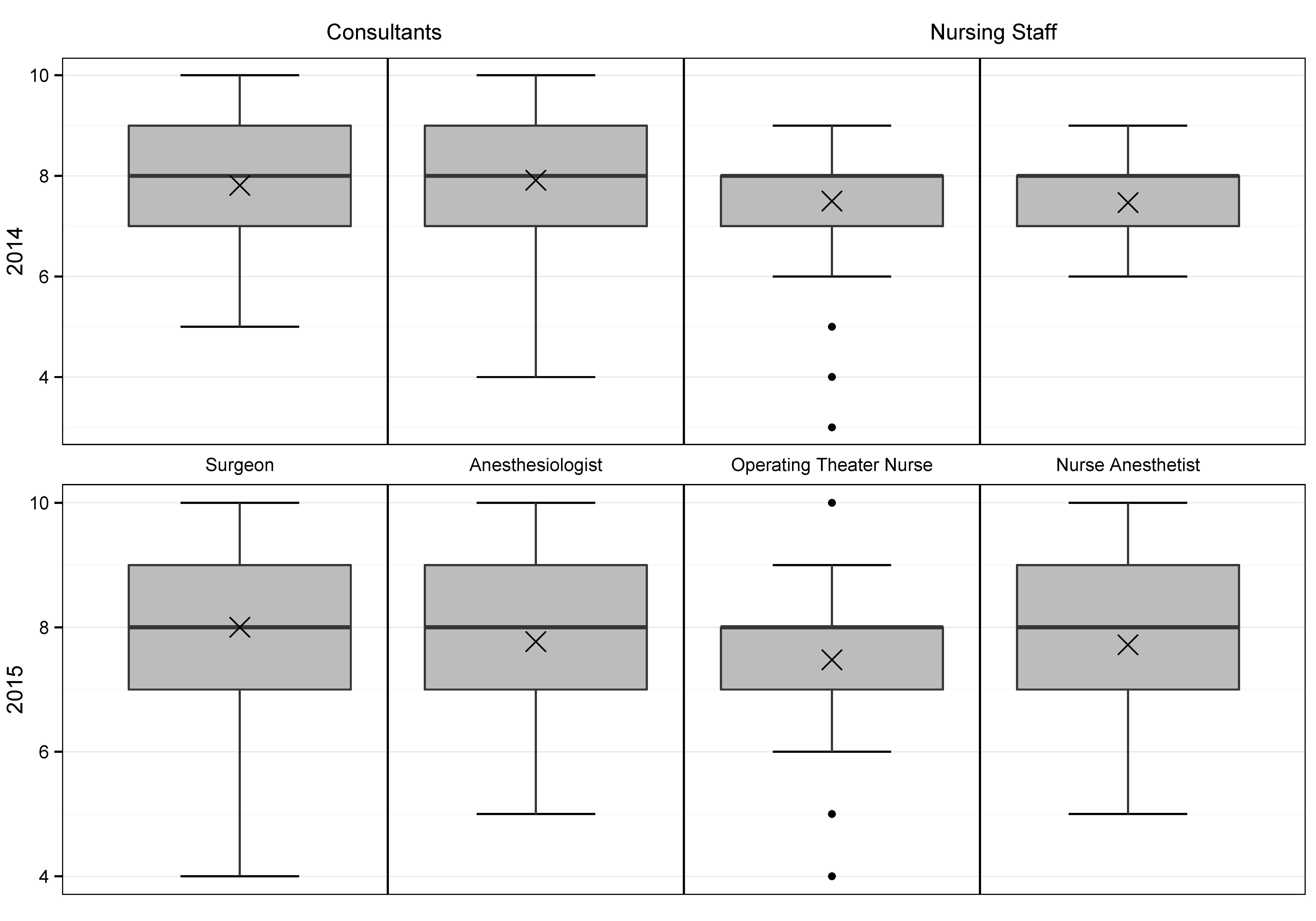

Supplement: S4 Fig — (TIF) [file pone.0149212.s004.tif]
