# Supplementary material for: The Gap between Individual Perception and Compliance: A Qualitative Follow-Up Study of the Surgical Safety Checklist Application
Source: PLoS One. 2016 Feb 29;11(2):e0149212. doi: 10.1371/journal.pone.0149212 (PMC4771169; doi:10.1371/journal.pone.0149212)
Supplement: S1 Table — Question 2 of objective knowledge was changed to adhere to local procedures. (DOCX) [file pone.0149212.s005.docx]

**S1 Table.** Questionnaire items: general use of the SSC, frequency of SSC use, satisfaction with the implementation, subjective and objective knowledge (correct answer in brackets) as well as individual perception of the SSC’s usefulness as used in the Swiss survey [26]. Question 2 of objective knowledge was changed to adhere to local procedures.

| **General question** | **Do you use a Surgical Safety Checklist to support patient safety?** (yes/no) |
| --- | --- |
| **Frequency of SSC use** | **In how many operations do you use the Surgical Safety Checklist at your primary working place?** |
|  | - Never or almost never (0-10% of operations) |
|  | - Rarely (11-30% of operations) |
|  | - Occasionally (31-50% of operations) |
|  | - Frequently (51-70% of operations) |
|  | - Most of the time (71-90% of operations) |
|  | - Always or almost always (91-100% of operations) |
| **Satisfaction with SSC** | **How satisfied are you with the implementation of the Surgical Safety Checklist at your primary working place?** |
|  | - Very satisfied |
|  | - Satisfied |
|  | - Somewhat satisfied |
|  | - Rather unsatisfied |
|  | - Very unsatisfied |
| **Subjective knowledge** | **How do you rank your knowledge with respect to the content and utilization of the Surgical Safety Checklist?** |
|  | - Very good |
|  | - Rather good |
|  | - Okay |
|  | - Rather bad |
|  | - Very bad |
| **Objective knowledge** | **Questions** |
|  | The SSC is a synonym for Team Time Out. (false) |
|  | The SSC must not be completed by all team members. (false) |
|  | The SSC requires exact documentation of the number of used sponges. (false) |
|  | The SSC exclusively addresses surgeons. (false) |
|  | The SSC recommends an antibiotic prophylaxis within 60 minutes of surgery. (true) |
|  | The SSC shall support inexperienced members of the team. (false) |
|  | The SSC is a tool used to attribute mistakes and misses to specific persons. (false) |
|  | The SSC aims to prevent accidental omissions within routine procedures. (true) |
|  | The SSC aims to improve team communication. (true) |
|  | The SSC may be used to document complications. (false) |
| **Individual perception** | **The use of the SSC is** (7-point-Likert-scale) |
|  | easy (=7) or difficult (=1) |
|  | comfortable (=7) or uncomfortable (=1) |
|  | familiar (=7) or unfamiliar (=1) |
|  | important (=7) or not (=1) |
|  | good (=7) for employees or not (=1) |
|  | good (=7) for patients or not (=1) |
| **Demographic Data** | age |
|  | female or male |
|  | professional experience |
|  | hours spent in the OR in an average week |
|  | profession |
