# Supplementary material for: The Gap between Individual Perception and Compliance: A Qualitative Follow-Up Study of the Surgical Safety Checklist Application
Source: PLoS One. 2016 Feb 29;11(2):e0149212. doi: 10.1371/journal.pone.0149212 (PMC4771169; doi:10.1371/journal.pone.0149212)
Supplement: S2 Table — (DOCX) [file pone.0149212.s006.docx]

**S2 Table.** Questionnaire items in the original language as used in the Swiss survey [26]: Wissen und Einstellung zur OP-Checkliste

| **Generelle Frage** | **Verwenden Sie derzeit eine OP-Checkliste zur Förderung der PatientInnensicherheit?** (Ja/Nein) |
| --- | --- |
| **Häufigkeit der Anwendung** | **Wie häufig wird die OP-Checkliste an Ihrem primären Arbeitsort eingesetzt?** |
|  | - Nie oder fast nie (0-10% der Eingriffe) |
|  | - Selten (11-30% der Eingriffe) |
|  | - Gelegentlich (31-50% der Eingriffe) |
|  | - Häufig (51-70% der Eingriffe) |
|  | - Meistens (71-90% der Eingriffe) |
|  | - Immer oder fast immer (91-100% der Eingriffe) |
| **Zufriedenheit mit Umsetzung** | **Wie zufrieden sind Si emit der Umsetzung der OP-Checkliste an Ihrem primären Arbeitsort?** |
|  | - Sehr zufrieden |
|  | - Zufrieden |
|  | - Einigermaßen zufrieden |
|  | - Eher unzufrieden |
|  | - Sehr unzufrieden |
| **Subjektives Wissen** | **Wie schätzen Sie Ihr Wissen zum Inhalt und zur Anwendung der OP-Checkliste ein?** |
|  | - Sehr gut |
|  | - Eher gut |
|  | - Einigermaßen |
|  | - Eher schlecht |
|  | - Sehr schlecht |
| **Objektive Wissen** | **Fragen** |
|  | Die OP-Checkliste ist eine andere Bezeichnung für Team Time Out. (falsch) |
|  | Die OP-Checkliste muss nicht von allen Teammitgliedern durchgeführt werden. (falsch) |
|  | Die OP-Checkliste erfordert die genaue Dokumentation der Anzahl der verwendeten Tupfer. (falsch) |
|  | Die OP-Checkliste richtet sich ausschließlich an ChirurgInnen. (falsch) |
|  | Die OP-Checkliste geht von einem Zeitfenster für die Antibiotikaprophylaxe von 60 Minuten aus. (richtig) |
|  | Die OP-Checkliste ist für unerfahrenes Personal gedacht. (falsch) |
|  | Mit der OP-Checkliste sollen Versäumnisse und Fehler einzelnen Personen zugeordnet werden. (falsch) |
|  | Mit der OP-Checkliste soll das versehentliche Auslassen von Routineabläufen verhindert werden. (richtig) |
|  | Mit der OP-Checkliste soll die Lommunikation im Team gefördert werden. (richtig) |
|  | Mit der OP-Checkliste sollen Komplikationen dokumentiert werden. (falsch) |
| **Beurteilung der OP-Checkliste** | **Die OP-Checkliste zu benutzen ist** (7-teilge-Likert-Skala) |
|  | einfach (=7) bis schwierig (=1) |
|  | angenehm (=7) bis unangenehm (=1) |
|  | gewohnt (=7) bis ungewohnt (=1) |
|  | wichtig (=7) bis unwichtig (=1) |
|  | Gut für Mitarbeitende (=7) bis schlecht für Mitarbeitende (=1) |
|  | Gut für PatientInnen (=7) bis schlecht für PatientInnen (=1) |
| Demographische Daten | Alter |
|  | Geschlecht |
|  | Wie lange üben Sie die Tätigkeit schon aus? |
|  | Wie viele Stunden pro Woche verbringen Sie am Patienten bzw. an der Patientin im OP? |
|  | Berufsgruppe |
